# Delayed superior epigastric artery pseudoaneurysm following percutaneous radiologic gastrostomy: Treatment by percutaneous embolization with N-butyl cyanoacrylate

**DOI:** 10.1016/j.radcr.2021.03.050

**Published:** 2021-04-12

**Authors:** Rémi Grange, Clément Chevalier-Meilland, Bertrand Le Roy, Sylvain Grange

**Affiliations:** a Department of Vascular Radiology, University Hospital of Saint-Etienne, Saint-Etienne, France; b Department of Digestive and Hepatobiliary Surgery, University Hospital of Saint-Etienne, Saint-Priest-en-Jarez, France

**Keywords:** Gastrostomy, Bleeding, Pseudoaneurysm, Embolization

## Abstract

Percutaneous radiologic gastrostomy (PRG) is a widely used procedure with a low rate of serious complications and with comparable short-term outcomes with percutaneous endoscopy. Hemorrhagic complications are rare (1.4%), and occur usually immediately after the procedure due to direct arterial punctures. We report on the case of a 62-year-old male patient with a diagnosis of multi-systemic atrophy disease that was referred to our tertiary center for PRG. The procedure was performed without early complications. He presented a slight bleeding 3 weeks of the procedure. A CT angiogram revealed a pseudoaneurysm of the left superior epigastric artery, in contact with the gastrostomy tube. After a failed surgical treatment, the patient was successfully treated by percutaneous embolization using a mixture of Glubran 2 and Lipiodol, under ultrasound and fluoroscopic control. This case study suggested that a slight hemorrhage following PRG may suggest a pseudoaneurysm and a CT angiogram should be performed.

## Introduction

Percutaneous radiologic gastrostomy (PRG) is a widely used method of enteral access for patients requiring nutritional support. This technique has high technical success rate [1], and low procedure-related mortality and is comparable to percutaneous endoscopic gastrostomy (PEG) in terms of mortality and complications [2,3]. Additionally, it can be performed under local anesthesia, and/or the passage of the gastroscope is impossible due to radiation or tumor stenosis. Hemorrhagic complications are very rare [4] and occur immediately due to direct arterial punctures [1]. We present a very rare case of slight bleeding, which occurred following a pseudoaneurysm of the superior epigastric artery.

## Case presentation

A 62-year-old male patient with advanced undernutrition caused by a multiple system atrophy for at least 3 years was referred to our tertiary center for PRG. The PRG procedure was performed under local anesthesia with fluoroscopic guidance after informed consent of the patient.

First, the absence of any obstacle in the left hepatic lobe was confirmed under ultrasound examination. Then, local anesthesia was administered with a 22G needle, from the skin to the stomach, with a mixture of 20ml of Lidocaine, Ropivacaine and Bicarbonate. Three anchors were then placed in order to achieve gastropexy. Finally, a 16F gastrostomy tube was placed, using the Seldinger technique.

**Fig. 1:**
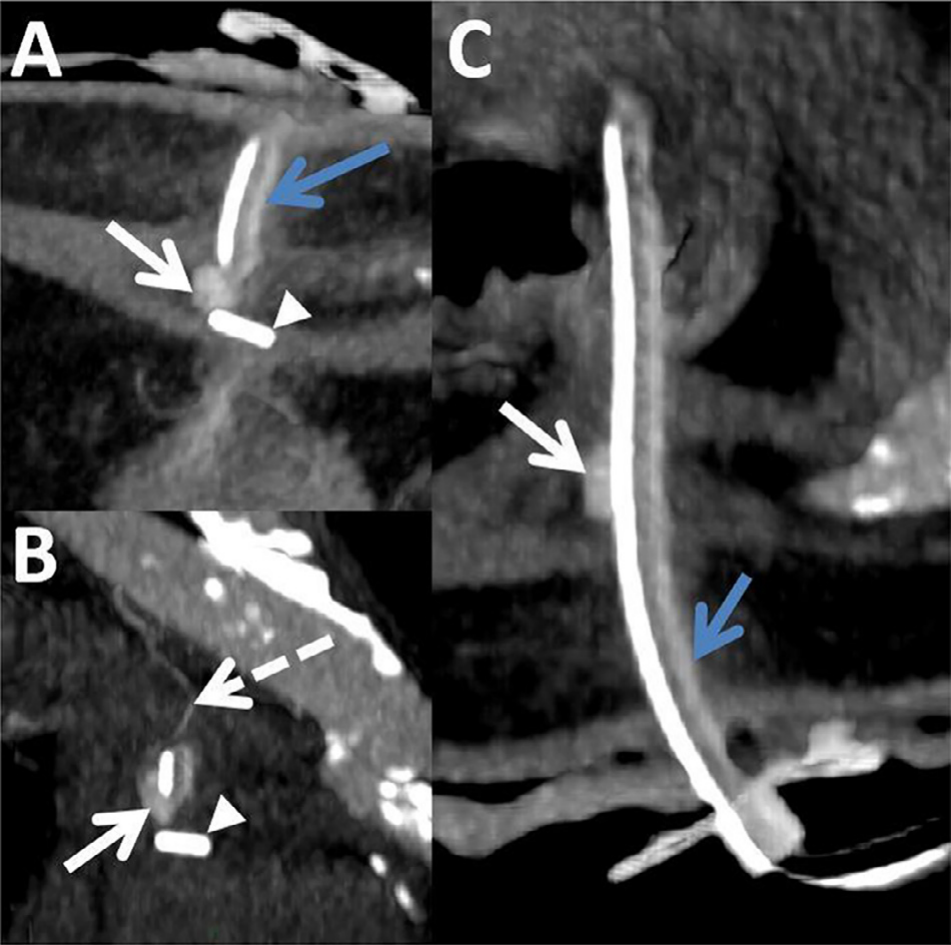
Abdominal CT scan injected at arterial time in axial (A), coronal (B) section and along the long axis of the gastrostomy tube (C), showing an addition image (white arrow) of 9mm compatible with a pseudoaneurysm, in contact with the gastrostomy tube (blue arrow) and the anchor (arrowhead), in the continuity of the upper epigastric artery (dotted arrow).

**Fig. 2:**
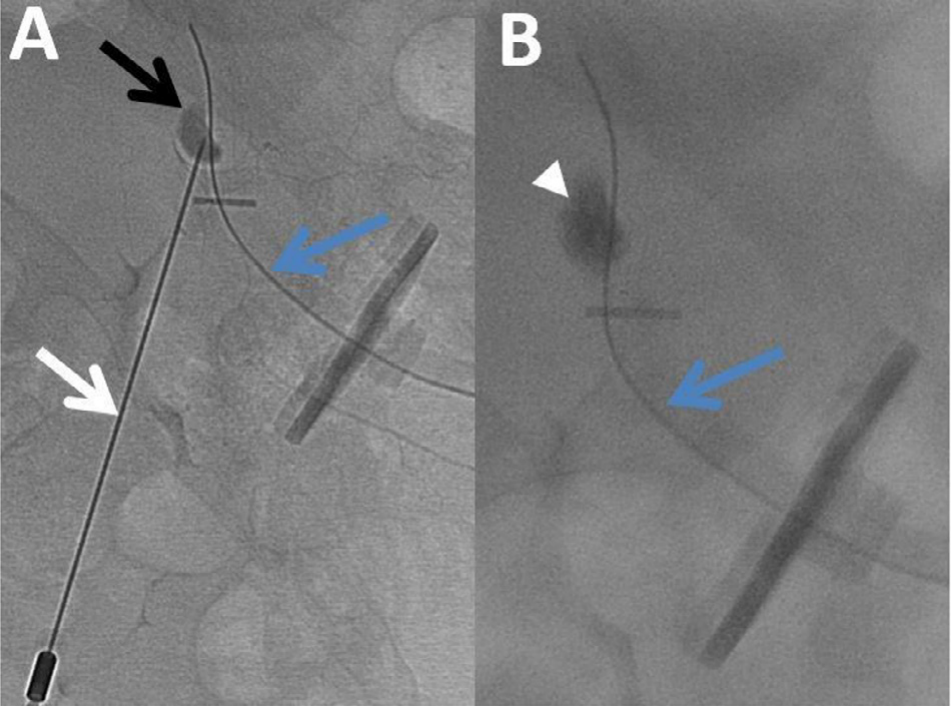
Fluoroscopic image (A) showing the opacification of the pseudoaneurysm (black arrow) by direct puncture with the 22G needle (white arrow) in contact with the gastrostomy tube (blue arrows). The fluoroscopic control image (B) shows the complete opacification of the aneurysm (arrowhead) with injection of a 0.5 ml mixture of Lipiodol and Glubran 2 (ratio 1/1).

**Fig 3:**
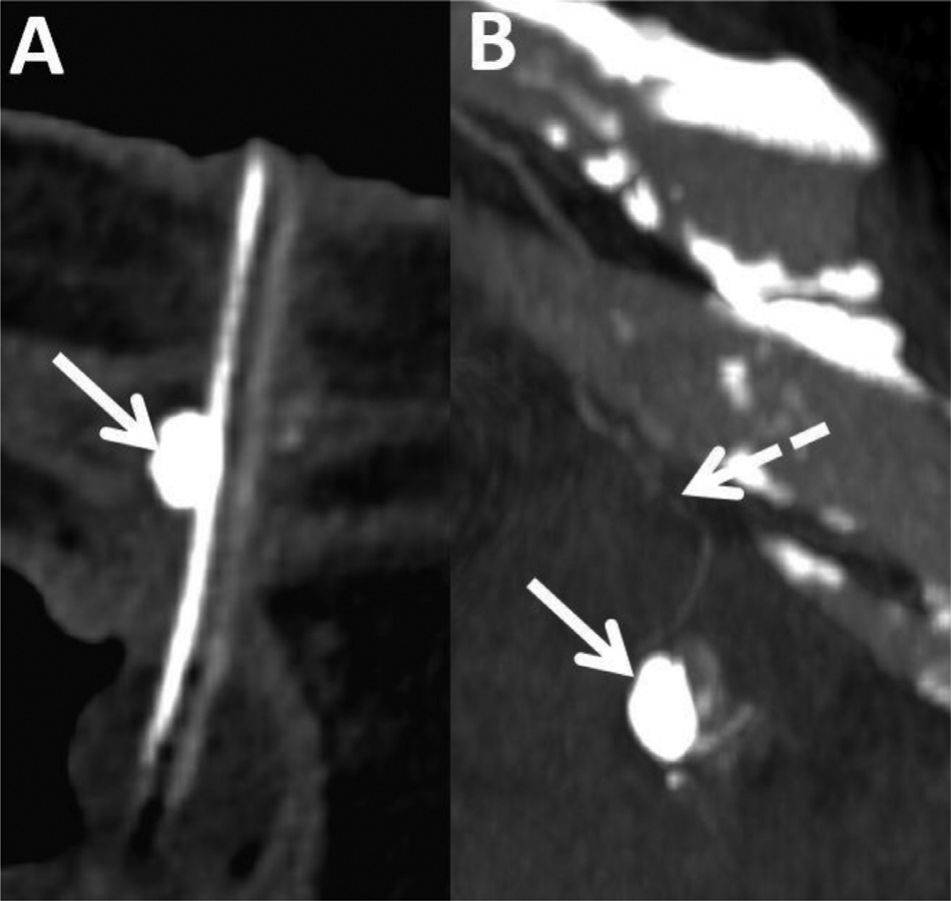
Abdominal CT scan without injection in axial section (A) confirms the complete filling of the pseudoaneurysm with the Lipiodol/Glubran2 mixture (white arrow). The coronal section scan (B) injected at arterial time shows the preserved permeability of the upper epigastric artery (dotted arrow).

The treatment was performed without early complications and the patient was transferred to the neurology department. Enteral nutrition was initiated 24 hours later. He was readmitted three weeks later for slight hemorrhagia, with a decrease of 5.4 hemoglobin points (from 12.9 to 7.5 g/dl) in 25 days, which required transfusion of two units of red blood cells. Initially, a surgical approach with cauterization under local anesthesia, in the peripheral area of the gastrostomy, was carried out. Subsequently, the bleeding persisted, requiring a computed tomography (CT) scan which showed a correct placement of the three anchors and the gastrostomy tube, without subcutaneous or intra-gastric hematoma (Fig. 1). A 9mm focal dilatation from the left superior epigastric artery, in contact with the gastrostomy tube and one of the three anchors was also vizualized. No active leakage of contrast agent was observed. It was compatible with a pseudoaneurysm. After a multidisciplinary meeting including surgeons and vascular radiologists, percutaneous embolization was decided. The patient was then transferred to the interventional radiology room. An ultrasound confirmed a pulsatile rounded image in color Doppler, into the left straight muscle of the abdomen, in contact with the left superior epigastric artery. First, the pseudoaneurysm was punctured under ultrasound guidance, using a 22G-needle. Under fluoroscopic guidance, iodine-contrast agent was injected, which allowed us to confirm the correct position of the needle. After flushing the needle with 5% dextrose solution, a mixture of 0.5ml of N-butyl cyanoacrylate (NBCA) (Glubran 2, GEM Srl, Viareggio, Italy) and ethiodized oil (Lipiodol, Villepinte, Guerbet, France) was injected in a 1:1 ratio, until complete opacification of the pseudoaneurysm (Fig. 2). The control CT scan showed the persistent permeability of the superior epigastric artery, the absence of contrast leakage, and non-opacification of the pseudoaneurysm (Fig. 3). The patient had no further bleeding and the remote control CT scan showed complete embolization of the pseudoaneurysm. The patient died 6 months later from his underlying disease.

## Discussion

To our knowledge, this is the first case report of a pseudoaneurysm of the superior epigastric artery following PRG, subsequently treated by percutaneous injection. PEG and PRG have comparable procedural-related mortality and complication rates (2.19% and 0.07% respectively), markedly lower than surgical procedures [5], [6]. Major complications include: abscess formation, aspiration pneumonia, bowel perforation, peritonitis, and haemorrhage. Hemorrhage is a result of direct puncturing of the left gastric [7], gastroepiploic [8], or epigastric arteries and occurs in 1.4% of patients [1]. In this case, we followed the recommendations to puncture through the upper third of the abdomen in order to avoid direct puncture of the epigastric artery [9]. Hemorrhages are usually treated by endoscopy, with ligation or sclerosis, or by endovascular embolization. Peptic ulcer disease, anticoagulation, and previous anatomic alterations are risk factors of haemorrhaging during PRG [8]. None of these risk factors were present in this patient.

In a retrospective observational study on 508 PRG procedures, de Baere and al. [7] reported one (0.2%) major immediate case of bleeding from the left gastric artery requiring embolization without case of delayed or low-noise bleeding. Delayed bleeding is in fact extremely rare after PRG. To our knowledge, a single delayed bleeding following a pseudoaneurysm of the gastro-epiploic artery, treated by endovascular approach, has been reported [8](ref). Superficial and visceral pseudoaneurysms are uncommon and associated with a high mortality if left untreated. They result from a breach in the vessel wall during an inflammatory, traumatic or tumor process, so that blood is only contained by the adventitia. Percutaneous approaches have been shown to be effective for the treatment of intraparietal or intra-abdominal pseudoaneurysms [10]. Such approaches are commonly reserved in case of failure of endovascular treatments due to anatomical factors or atherosclerotic changes. However, they can in some cases be performed as a first-line treatment, under CT or fluoroscopic/ultrasound guidance [10], depending on the location, the size of the pseudoaneurysm, and the experience of the operator.

Compared to endovascular procedure, percutaneous procedure represent a less expensive, time-saving approach and limits irradiation and injection of iodinated contrast agent [10]. The small size of the aneurysm was not a limiting factor in this case, given the superficial location of the pseudoaneurysm. The use of a thin 22G needle allowed us to bypass digestive and vascular structures without risk of injury, but exposes to the risk of sticking the needle during injection. In this case, we decided to make a highly viscous mixture of NBCA/Lipiodol because of the small volume of the aneurysmal sac and the risk of non-target embolization.

In conclusion, this case showed a very rare case of delayed bleeding following a pseudoaneurysm of the superior epigastric artery after PRG. Slight bleeding following PRG should be checked for pseudoaneurysm by use of a contract-enhanced CT scan. Percutaneous embolization appears to be a safe and effective approach to treat pseudoaneurysms of the epigastric artery. This could be performed as first-line treatment when pseudoaneurysm is visible under ultrasound.

## Patient consent

The patient's consent could not be obtained before his death. The authors declare that this report does not contain any personal information that could lead to the identification of the patient.
